# Safety and efficacy of tocilizumab treatment in children with systemic onset of juvenile idiopathic arthritis

**DOI:** 10.1186/1546-0096-9-S1-P202

**Published:** 2011-09-14

**Authors:** E Alexeeva, R Denisova, S Valieva, T Bzarova, K Isayeva, T Sleptsova, E Mitenko

**Affiliations:** 1 Scientific Center of Children's Health, Moscow, Russian Federation

## Objectives

To evaluate safety and efficacy of tocilizumab treatment in children with systemic onset of juvenile idiopathic arthritis (JIA).

## Methods

A retrospective observational study on JIA patients taking tocilizumab (n=39). Tocilizumab was administered intravenously at a dose of 8 mg/kg every 2 weeks during 2 months then every 4 weeks. All patients received DMARDs. Efficacy end points included the American College of Rheumatology (ACR) Pediatric 30 (Pedi 30), Pedi 50, Pedi 70, and Pedi 90 criteria for improvement.

## Results

A total of 39 patients (21 boys and 18 girls) were included in this Median age was 7,5 years (range; 3 to 15 years) and median disease duration was 4,2 years (range; 0.5 to 8,3 years). A total of 16 of the 39 patients (25%) entered 52 weeks of continuous tocilizumab treatment. The frequently observed non-severe adverse events were nasopharyngitis, upper respiratory tract infection and gastroenteritis. No cases of opportunistic infections, malignancies, autoimmune diseases, or death were reported. One case of pneumonia. 21 patients had incidences of neutropenia.The ACR Pedi 30, 50, 70 and 90 were achieved by 82%,50%, 27% and 12% of patients at Week 4 (N=36), and by 100%, 81%, 69%, and 50% of patients at Week 24 (N=18), and by 100%, 85%, 78%, and 57% of patients at Week 52 (N=16), respectively.

## Conclusion

Clinical improvements in the signs and symptoms of systemic JIA were also achieved in favorable levels in tocilizumab in the treatment of children with JIA.

